# Dielectric Properties of Hybrid Polyethylene Composites Containing Cobalt Nanoparticles and Carbon Nanotubes

**DOI:** 10.3390/ma15051876

**Published:** 2022-03-02

**Authors:** Ieva Vanskevičė, Mariya A. Kazakova, Jan Macutkevic, Nina V. Semikolenova, Juras Banys

**Affiliations:** 1 Faculty of Physics, Vilnius University, 10222 Vilnius, Lithuania; i.kranauskaite@yahoo.com (I.V.); juras.banys@ff.vu.lt (J.B.); 2 Boreskov Institute of Catalysis, SB RAS, Lavrentieva 5, 630090 Novosibirsk, Russia; manj86@mail.ru (M.A.K.); nvsemiko@catalysis.ru (N.V.S.)

**Keywords:** dielectric permittivity, cobalt nanoparticles, carbon nanotubes

## Abstract

Polymer composites with electrically conductive inclusions are intensively developed for microwave shielding applications, where lightweight and elastic coatings are necessary. In this paper, dielectric properties of hybrid polyethylene composites containing cobalt nanoparticles and multi-wall carbon nanotubes (MWCNT) were investigated in the wide frequency range of 20–40 GHz for electromagnetic shielding applications. The percolation threshold in the hybrid system is close to 6.95 wt% MWCNT and 0.56 Co wt%. Cobalt nanoparticles (up to highest investigated concentration 4.8 wt%) had no impact on the percolation threshold, and for the fixed total concentration of fillers, the complex dielectric permittivity is higher for composites with bigger MWCNT concentrations. Moreover, the microwave complex dielectric permittivity of composites with high concentration of fillers is quite high (for composites with 13.4 wt% MWCNT and 1.1 wt% Co ε′ ≈ ε″ ≈ 20 at 30 GHz, it corresponds to microwave absorption 50% of 1 mm thickness plate); therefore, these composites are suitable for electromagnetic shielding applications.

## 1. Introduction

Fast progress of telecommunications and electronics leads to the broad use of electromagnetic waves in various areas. The issues of electromagnetic interference become principal since various apparatuses work in the same or neighbor frequency ranges and are due to the generation of electromagnetic waves by some natural phenomena (for example lightning); moreover, the power of electromagnetic waves increases with the number of apparatuses working in the same place and by the fast growing industry of telecommunications, where higher frequencies and herewith higher power are needed for the transference of a larger quantity of information [1,2]. The electromagnetic interference can cause damages to electronic devices and can be harmful for human health [3]. In addition, the electromagnetic waves can be used for unsanctioned information collecting or tracking of peoples or vehicles. Traditional electromagnetic shielding materials involve highly electrically conductive metals such as Al, Cu [4]. The main drawbacks of metals for such applications are the high density, easy corrosion, poor flexibility and big reflected part of electromagnetic radiation. In contrast, coatings based on polymer composites filled with various electrically conductive inclusions are lightweight, easy processable and chemically inert [5].

Composites with various carbon nanoinclusions (single-wall carbon nanotubes (SWCNT), multi-wall carbon nanotubes (MWCNT), carbon black, graphene and onion-like carbon) have been widely investigated for electromagnetic shielding and electrical conductivity applications [6,7,8]. Indeed, such composites exhibit low percolation thresholds (the lowest concentrations at which composites are electrically conductive) and big electromagnetic shielding at concentrations substantially higher than the percolation threshold [9,10]. The main drawback of composites with carbon nanoinclusions is that often nanoparticles inside the polymer matrix form bigger or smaller clusters and, therefore, the electromagnetic properties of composites are determined by clusters of nanoparticles [11]. To overcome this drawback, other types of nanoparticles can be added to the composites matrix. Indeed, it was demonstrated that the addition of insulating MgO nanoparticles can change the electrical conductivity of composites with MWCNT by several orders [12]. Moreover, the addition of magnetic nanoparticles to composites with carbon nanoinclusions can cause additional electromagnetic shielding due to magnetic losses [13]. For composites with several nanoparticle types, the synergy effect becomes important, which is the enhancement of composite properties due to interactions of nanoparticles inside the polymer matrix [14,15]. For example, in hybrid composites, the percolation threshold can be substantially lower, while electromagnetic properties are significantly better than in composites with one type inclusions [16]. Cobalt nanoparticles are widely used for electromagnetic shielding applications [17,18,19]. However, electrical conductivity values of polymeric composites with cobalt nanoinclusions are not big enough [19] and the addition of other more conductive nanoparticles is required to reach the sufficient electromagnetic shielding [20,21]. However, the impact of cobalt nanoparticles on broadband dielectric properties of composites with MWCNT in wide temperatures is still unknown.

The aim of this paper is to investigate broadband dielectric properties of polyethylene composites with MWCNT and Co nanoparticles in a wide temperature range. Investigations were performed for composites with relatively low Co nanoparticles concentrations (not bigger as 4.8 wt%); at these concentrations, the electrical percolation in composites with cobalt nanoparticles is not expected [22], and various MWCNT concentrations range up to 13. 6 wt%.

## 2. Materials and Methods

### 2.1. Co/MWCNT-PE Composites Preparation

The Co/MWCNT-PE composites preparation procedure consisted of two main stages. Initially, Co/MWCNT hybrids were prepared with various cobalt content. The MWCNTs used for preparation of Co/MWCNT were synthesized by chemical vapor deposition (CVD) of ethylene gas over bimetallic Fe-Co catalysts at 680 °C [23]. The obtained nanotubes had a wall number of 12–15 and an average outer and inner diameter of 9.4 and 4 nm, correspondingly. The characteristics of the used MWCNTs are described in more detail in [24,25]. After the growth, the MWCNT were purified from catalyst particles by boiling in a 15% solution of hydrochloric acid. Subsequently, the MWCNT were oxidized in concentrated nitric acid for 2 h to obtain functionalized nanotubes with about 2.4 carboxylic groups per nm^2^, a surface area of 260 m^2^ g^−1^ and a purity above 99% [26]. Co/MWCNT samples containing from 3.5 to 14.5 wt% of cobalt were prepared by a one-step incipient wetness impregnation with cobalt nitrate solutions (Co(NO_3_)_2_ × 6H_2_O, 98%, Sigma-Aldrich (St. Louis, MO, USA) with a required concentration. In order to obtain samples with a high Co content, multiple sequential impregnation was used. For this, a 7.5 wt% Co/MWCNT sample was impregnated with aqueous solutions of cobalt nitrate, followed by drying at 110 °C and re-impregnation until the required cobalt content was obtained, taking into account that about 7.5 wt% of Co was added in one impregnation. Depending on the number of impregnations, Co/MWCNT samples were obtained with a cobalt content from 14.1 to 51 wt%. Then, all the samples obtained were calcined at a temperature of 350 °C for 3 h, and after that, they were reduced in a flow of hydrogen at a temperature of 350 °C for 4 h. After cooling, the reduced samples were purged with argon for 20 min and transferred into glass ampoules, which were sealed without contact with air immediately after the reduction procedure. The obtained samples were stored in glass ampoules until the polymerization procedure. The procedure for the synthesis of Co/MWCNT hybrids is described in more detail in [20,21,26]. The exact Co content was determined by X-ray fluorescence (XRF) using an ARL Perform’X (Termo Fisher Scientific, Waltham, MA, USA) sequential X-ray tube spectrometer with Rh anode [27]. The obtained samples were denoted as follows: x Co/MWCNT-Ox, where x reflects the cobalt content in wt%.

The synthesis of Co/MWCNT-PE composites was carried out using an in situ polymerization technique based on the preliminary adsorption of a Ti-containing polymerization catalyst on the surface of the Co/MWCNTs hybrids followed by ethylene polymerization. All procedures for the preparation of composites were carried out in an argon atmosphere according to the standard Schlenk techniques. The in situ polymerization technique and the technology for preparing Co/MWCNT-PE composites are described in more detail in [21,28,29]. The polymerization reaction was carried out to obtain a composite material containing about 10 wt% of x%Co/MWCNT additive. The resulting product was separated from the reaction mixture, washed with heptane and ethanol and dried to constant weight. Thus, powders of Co/MWCNT-PE composites with different ratios of Co:MWCNT:PE components were obtained.

The composites were denoted as y% (x% Co/MWCNT), where y% refers to the content (x% Co/MWCNT) of the hybrid (i.e., total fillers concentration) in the composite, and x% to the Co content in the hybrid, where y-x is the mass concentration of MWCNT in wt%. Investigated composites photos are presented in Figure S1.

### 2.2. Structure Characterization of Co/MWCNT-PE Composites

The structure of the x% Co/MWCNT hybrids was characterized by transmission electron microscopy (TEM) on a JEOL JEM-2010 microscope (Akishima, Tokyo, Japan) operating at an accelerating voltage of 200 kV, which provides a nominal resolution of 0.14 nm. The Co particle size distribution in Co/MWCNT hybrids was estimated by analyzing TEM images containing about 200–400 Co particles at ×50,000 and ×400,000 magnifications.

The morphology of the Co/MWCNT hybrids and Co/MWCNT-PE composites, as well as the homogeneity of distribution of Co/MWCNT hybrids in a polyethylene matrix, were characterized by scanning electron microscopy (SEM) using a JSM6460LV JEOL microscope (Akishima, Tokyo, Japan) with an accelerating voltage of 25 kV. Hot pressed composite films were used for SEM studies as described in Section 2.3 below. The composite films were cut into pieces of 8 × 3 × 0.5 mm^3^ and then attached with silver glue to a copper support so that the cut-out section was facing the beam.

### 2.3. Electrophysical Characteristics of Co/MWCNT-PE Composites

To study the electrical properties of Co/MWCNT-PE composites, films prepared from the obtained powders of composite materials by hot pressing using a hand press between two polished steel plates covered with a Teflon film and a 0.5 mm thick copper frame were used.

In the frequency range from 20 Hz to 1 MHz, investigations were done using an LCR meter (HP4284) (Hewlett-Packard, Palo Alto, CA, USA) measuring the capacitance and the loss tangent. Measurements were performed on cooling from 300 K down to 30 K. In the frequency range from 1 MHz to 3 GHz, measurements were performed by coaxial dielectric spectrometer with a vector network analyzer (Agilent 8714ET) (Agilent Technologies, Santa Clara, CA, USA) by measuring the complex reflection coefficient. The silver paste was used for contacts.

Microwave measurements (27 GHz–40 GHz) were carried out with a scalar network analyzer (R2-408R, ELMIKA, Vilnius, Lithuania) measuring complex reflection and transmission modules.

## 3. Results

### 3.1. Structure of Composites

As it is known, the electrophysical characteristics of composite materials are influenced by various factors, including the composition of the composite material, as well as the uniformity of additive distribution in its composition. The way fillers are introduced into the polymer is a decisive factor affecting their distribution and the properties of the final product. Previously, we carried out a comparative study of the influence of the homogeneity of MWCNT distribution on the electrical properties of MWCNT-polyethylene composites obtained by mechanical mixing in a polymer melt, coagulation precipitation and in situ polymerization of ethylene using a Ziegler-Natta catalyst supported on MWCNT. It has been shown that in situ polymerization produces composite materials with a more uniform distribution of MWCNT in the PE matrix as compared to other methods. The study of the electrophysical properties of the composites showed that the uniform distribution of MWCNTs in polyethylene provides high values of conductivity, which correlate with high values of the complex dielectric permittivity [30].

It should be noted that obtaining excellent electromagnetic interference shielding performance of polymer composites requires achieving a uniform distribution (which can be determined by various techniques, for example SEM [12]) of both dielectric and magnetic fillers in their composition. In this work, a uniform distribution of Co/MWCNT hybrids was provided using the in situ polymerization technique, whereas a uniform distribution of cobalt nanoparticles was achieved by using oxidized MWCNTs, which are characterized not only by hydrophilic surface properties, but also by the presence of structural defects in the walls that serve as centers for nanoparticles fixation [26].

In our previous studies, it was shown that varying the cobalt content in Co/MWCNT hybrids from 3.5 to 14.5 wt% leads to an increase in the particle size on the MWCNT surface, as well as a redistribution of the cobalt particles population in the MWCNT (inner channels/surface) [20,21]. Thus, in the case of a sample containing 3.5 wt% of cobalt, all nanoparticles are localized in the internal channels of MWCNTs, while for a sample containing 14.5 wt% Co, the population of particles in the internal channels is only 20% (Figure S2). A further increase in cobalt content leads to the formation of larger particles predominantly on the MWCNT surface (Figure S3). SEM micrographs of Co/MWCNT hybrids with a cobalt content of 21 to 51 wt% are shown in Figure S4. It should be noted that in samples with a high cobalt content, the formation of metal nanoparticles occurs not only on the surface of single MWCNT, but also in internodes formed by the interweaving of individual MWCNTs (Figure S4c,d).

**Figure 1:**
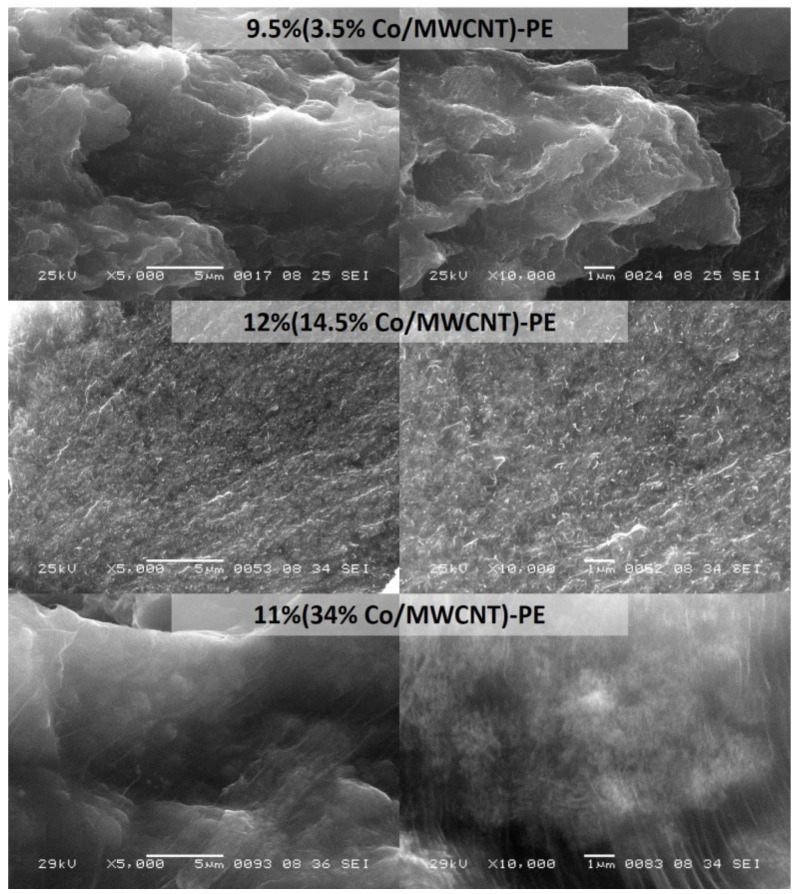
SEM images of composite materials at various magnification: 9.5% (3.5% Co/MWCNT)-PE, 12% (14.5% Co/MWCNT)-PE and 11% (34% Co/MWCNT)-PE.

The uniformity of Co/MWCNT hybrids distribution in the resulting composites was assessed using SEM (Figure 1). Analysis of SEM images of sufficiently low magnification allows us to see individual MWCNTs and their possible aggregates (visible as white “worms”); however, cobalt nanoparticles are not visible in Figure 1 due their size (Figures S2–S4). On the other hand, MWCNT covers a significant cross-sectional area of the composite. Analysis of the obtained images showed a uniform distribution of nanotubes over the cross section and the absence of MWCNT aggregates on various fillers concentrations (Figure 1).

### 3.2. Dielectric Properties at Room Temperature

**Figure 2:**
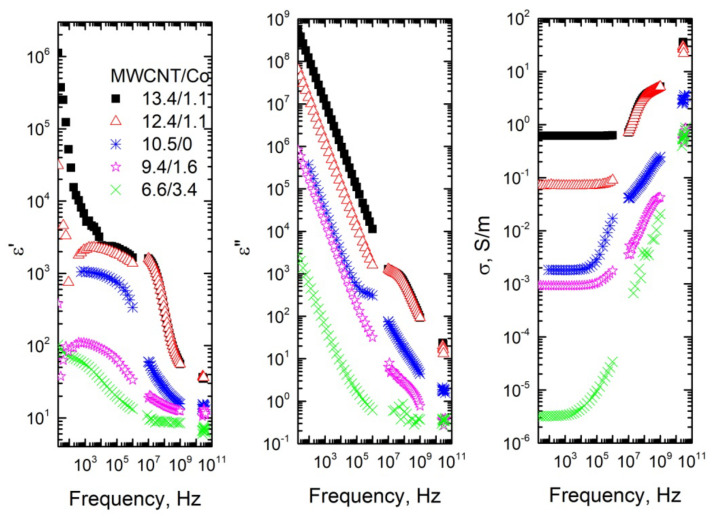
Frequency dependence of the real and the imaginary part of the complex dielectric permittivity and electrical conductivity for composites with various fillers concentrations.

**Figure 3:**
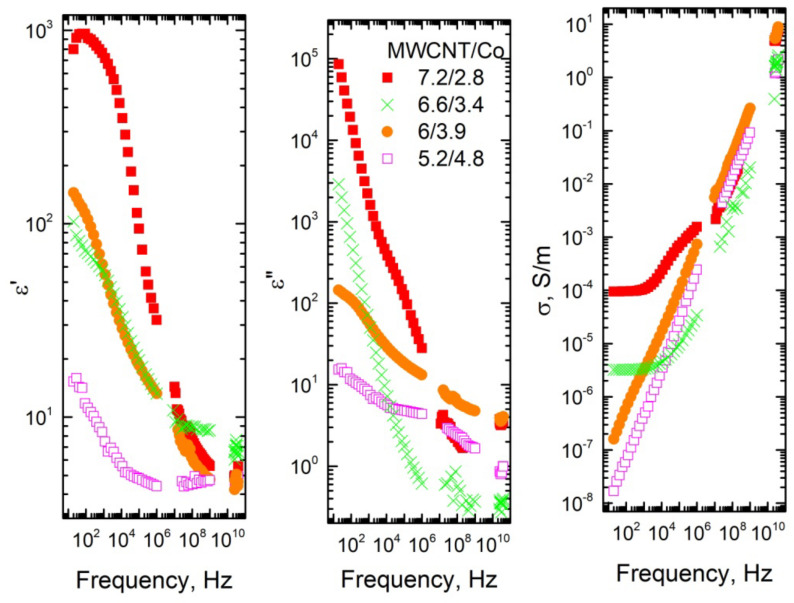
Frequency dependence of the real and the imaginary part of the dielectric permittivity and electrical conductivity for composites with total filler concentration 10 and 9.5 wt%.

The frequency dependencies of dielectric permittivity ε′, dielectric losses ε″ and electrical conductivity σ of composites with MWCNT/Co inclusions are presented in Figure 2 and Figure 3. The dielectric permittivity and the electrical conductivity increases with MWCNT concentration and together decreases with Co concentration in a wide frequency range for the same total filler concentration of 10 wt% (Figure 3). The dielectric permittivity and the electrical conductivity of composites with 5.2 wt% of MWCNT and 4.8 wt% of Co are low enough, and no frequency independent DC conductivity is observed in the conductivity spectra. Therefore, these composites are below percolation. In contrast, for composites with 7.2 wt% MWCNT and 2.8 Co wt%, the frequency of the independent conductivity plateau is observed. The similar conductivity plateau is observed for composites with 6.95 wt% of MWCNT and 0.56 wt% of Co inclusions (Table 1). Therefore, it can be concluded that the percolation threshold is close to 6.95 wt% of MWCNT and 0.56 wt% of Co in the system, while Co addition did not have an impact on the electrical percolation. Moreover, the impact of nanoparticles is expressed more at lower frequencies (below 1 MHz). It also should be admitted that, in our composites, the concentration of the Co nanoparticles is much smaller than the electrical percolation in composites with only these nanoparticles [22,31]. At such small Co concentrations, the dielectric permittivity of the composites is very small [22,31].

The values of the complex dielectric permittivity of the composites with a high concentration of MWCNT (above percolation threshold) in microwaves is quite high (for example, for composites with 13.4 wt% MWCNT and 1.1 wt% Co ε′ ≈ ε″ ≈ 20 at 30 GHz, it corresponds to microwave absorption 50% of 1 mm thick plate in the waveguide [32]) (Figure 2); therefore, these composites are suitable for electromagnetic applications. The electromagnetic properties of these composites in microwaves are comparable with the best properties of the composites published in literature [32,33]. The dielectric spectra of all investigated composites can be explained by Maxwell–Wagner polarization [34].

The electrical conductivity of composites above the percolation threshold can be described as frequency independent, *DC* conductivity and frequency dependent *AC* conductivity, determined by Jonscher‘s power law [35] (Figure 2 and Figure 3):

(1) $$\sigma =\sigma_{DC}+A\omega^{s}=\sigma_{DC}+{\left(\omega /\omega_{H}\right)}^{s}$$

where $\sigma_{DC}$ is the *DC* conductivity, *A* is the pre-exponental constant, ω is the angular frequency, *ω_H_* is the hopping frequency and *s* is the power law exponent, where 0 < *s* < 1 [36]. The critical frequency *f_cr_* was determined as the frequency at which the AC conductivity becomes 10% higher that the low frequency conductivity plateau (*DC*) value. The obtained parameters are summarized in Table 1.

All fit parameters non-monotonically depend on the total filler concentration. However, $\sigma_{DC}$, *f_cr_* and *ω_H_* correlate with each other, for bigger *DC* conductivity values are observed bigger *f_cr_* and *ω_H_* values [16]. For the fixed total filler concentration, values of abovementioned parameters decrease when the Co nanoparticles concentration increases. Values of *s* parameters are typical for hopping conductivity [36].

### 3.3. Electrical Conductivity at Low Temperatures

**Figure 4:**
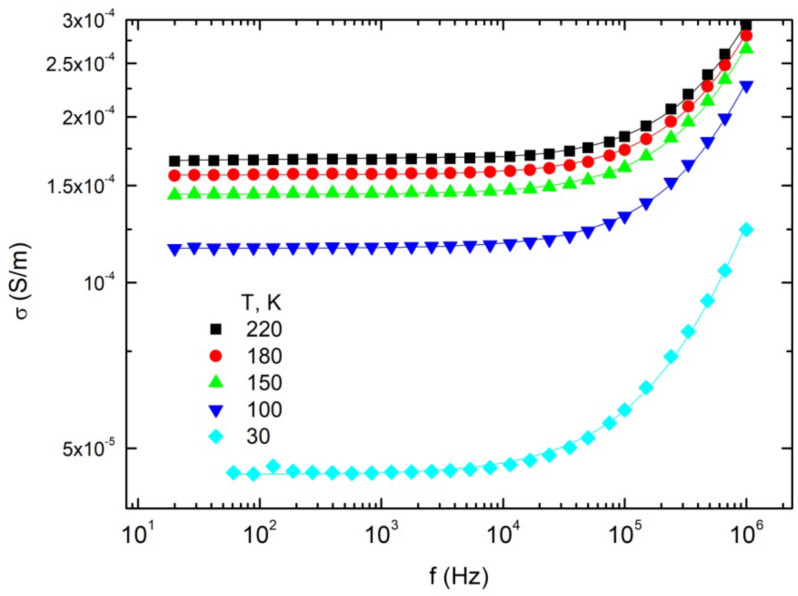
Frequency dependencies of electrical conductivity at various temperatures of composite with 9.4 wt%. MWCNT and 1.6 wt% Co nanoparticles. Solid lines are fits of Jonscher‘s power law (Equation (1)).

In Figure 4, it is clearly observed that on cooling the *DC* conductivity strongly decreases. On cooling, the critical frequency also decreases.

**Figure 5:**
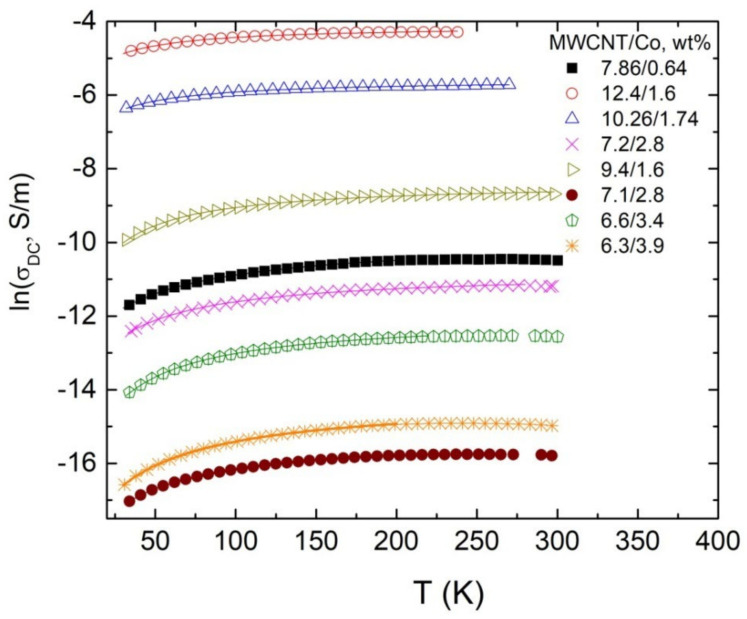
Temperature dependence of DC conductivity for composites. Solid lines are fits of the tunneling model. Numbers indicate *T_1_/T_0_* values and total concentration of the filler particles.

Temperature dependencies of the *DC* conductivity of various composites are presented in Figure 5. These dependencies fit the electrical tunneling model [37]:

(2) $$\sigma_{DC}=\sigma_{0}exp\left[\frac{T_{1}}{k\left(T+T_{0}\right)}\right],$$

where $T_{1}$ is the energy for an electron to cross the insulator gap between the conductive particles, and $T_{0}$ is the temperature above which thermally activated conductivity over the barrier occurs. The obtained tunneling model fit parameters are listed in Table 1. Only at low fillers concentrations (i.e., 6.6 wt% MWCNT and 3.4 wt% Co, 6.3 wt% MWCNT and 3.9 wt% Co) are discrepancies observed from the tunneling model fit, i.e., an increase of the electrical conductivity on cooling due to the rapid shrinkage of polyethylene above the glass transition temperature [38]. The tunneling model parameters $T_{1}$ and $T_{0}$ are related with microscopic parameters:

(3) $$T_{1}=wA\beta_{0}/8\pi k,$$

(4) $$T_{0}=2T_{1}/\pi \chi ,$$

where $\chi ={\left(2mV_{0}\right)}^{0.5}/h$ and $\beta_{0}=V_{0}/ew$; m and e are the electron mass and the charge, respectively; $V_{0}$ is the potential barrier amplitude; w is the interparticle distance (gap width) and A is the area of capacitance formed by the junction. From Equations (3) and (4), it follows that $T_{1}$/$T_{0}$ is proportional to the gap width w and the potential barrier $V_{0}$ amplitude. Tunneling fit parameters are presented in Table 2. $T_{1}$/$T_{0}$ decreases with the increase of the total concentration of filler particles and the total electric conductivity σ. Thus, the distance between the particles is decreasing with its total concentration. However, for composites with the same total concentration of fillers 10% MWCNT/Co, the ratio *T_1_/T_0_* is lower for composites with a bigger MWCNT concentration. Therefore, Co nanoparticles increase the potential barrier for electron tunneling between MWCNT clusters.

## 4. Conclusions

Dielectric properties of hybrid polyethylene composites containing cobalt nanoparticles and carbon nanotubes were investigated in wide frequency range 20–40 GHz. It was determined that the percolation threshold in the hybrid system is close to 6.95 wt% MWCNT and 0.56 wt% Co. Cobalt nanoparticles had no impact on the percolation threshold and, for the same total concentration of fillers, the complex dielectric permittivity is higher for composites with a bigger MWCNT concentration. The microwave complex dielectric permittivity of composites with a high concentration of fillers is quite high (for composites with 13.6 wt% MWCNT and 1.1 wt% Co ε′ ≈ ε″ ≈ 20 at 30 GHz, it corresponds to microwave absorption 50%); therefore, these composites are suitable for electromagnetic shielding applications.

## Figures and Tables

**Table 1:** Jonscher‘s power law fit parameters of conductivity spectra for composites above the percolation threshold and at room temperature.

Total Concentration/MWCNT/Co, wt%	*σ_DC_*	*f_cr_*	*ω_H_*, THz	*s*
7.5/6.95/0.56	0.24 mS/m	42 kHz	5.64	0.92
8.5/7.86/0.64	0.17 mS/m	2.7 kHz	32.8	0.82
9.5/9.2/0.3	0.21 S/m	2.39 MHz	175.7	1.04
9.5/7.5/2	0.04 µS/m	1.4 kHz	0.06	0.74
9.7/9.7/0	0.55 mS/m	17.7 kHz	251.2	1.05
9.9/6/3.9	2.2 µS/m	0.16 kHz	0.06	0.67
10/7.2/2.8	97.6 µS/m	1.2 kHz	102.7	0.91
10/6.6/3.4	3.19 µS/m	2.43 kHz	0.22	0.73
10.5/10.5/0	2.1 mS/m	37.2 kHz	995.7	1.26
11/9.4/1.6	0.92 mS/m	91.2 kHz	24.4	0.99
11.5/11.5/0	20 mS/m	356.6 kHz	1265	1.49
12/11.1/0.9	46.6 mS/m	596 kHz	832.9	1.32
14/12.4/1.6	71 mS/m	459.7 kHz	859.3	1.16
14.5/13.4/1.1	0.62 S/m	4.01 MHz	476.9	1.06
14.7/13.6/1.1	2.1 mS/m	470.7 kHz	556.6	1.84

**Table 2:** Tunneling model fit parameters.

MWCNT Concentration/Co Concentration, wt%	Ln {*σ*_0_, *S*/*m*}	*T*_1_, K	*T*_0_, K	*T*_1_/*T*_0_
7.86/0.64	−9.89	136	43	3.13
10.26/1.74	−5.57	41	20	2.05
12.4/1.6	−4.11	37	19	1.9
7.2/2.8	−10.78	109	32	3.41
9.4/1.6	−8.37	100	28	3.57
7.1/2.8	−15.28	114	31	3.68
6.6/3.4	−12.03	123	26	4.73
6/3.9	−14.27	154	36	4.72

## Data Availability

Data sharing is not applicable for this paper.

## Supplementary Materials

The following supporting information can be downloaded at: https://www.mdpi.com/article/10.3390/ma15051876/s1, Figure S1: Investigated composites photos., Figure S2: TEM images and Co nanoparticles size distribution in 3.5–14.5% Co/MWCNT hybrids (a–d) prepared by one step incipient wetness impregnation., Figure S3: TEM images and Co nanoparticles size distribution in 28–34% Co/MWCNT hybrids (a,b) prepared by multiple incipient wetness impregnation, Figure S4: SEM images for 21–51% Co/MWCNT hybrids (a,b) prepared by multiple incipient wetness impregnation.

## References

[1] Chung D.D.L. (2001). Electromagnetic interference shielding effectiveness of carbon materials. Carbon.

[2] Chen Z.P., Xu C., Ma C.Q., Ren W.C., Cheng H.M. (2013). Lightweight and flexible graphene foam composites for high-performance electromagnetic interference shielding. Adv. Mater..

[3] Van der Togt R., van Lieshout E.J., Hensbroek R., Beinat E., Binnekade J.M., Bakker P.J.M. (2008). Electromagnetic interference from radio frequency identification inducing potentially hazardous incidents in critical care medical equipment. JAMA-J. Am. Med. Assoc..

[4] Gonzalez M., Pozuelo C., Baselga J. (2018). Electromagnetic shielding materials in GHz range. Chem. Rec..

[5] Al-Saleh M.H., Saadeh W.H., Sundararaj U. (2013). EMI shielding effectiveness of carbon based nanostructured polymeric materials: A comparative study. Carbon.

[6] Al-Saleh M.H., Sundararaj U. (2009). Electromagnetic interference shielding mechanisms of CNT/polymer composites. Carbon.

[7] Qin F., Brosseau C. (2012). A review and analysis of microwave absorption in polymer composites filled with carbonaceous particles. J. Appl. Phys..

[8] Liang J.J., Wang Y., Huang Y., Ma Y.F., Liu Z.F., Cai J.M., Zhang C.D., Gao H.J., Chen Y.S. (2009). Electromagnetic interference shielding of graphene/epoxy composites. Carbon.

[9] Sandler J.K.W., Kirk J.E., Kinloch I.A., Shaffer M.S.P., Windle A.H. (2003). Ultra-low electrical percolation threshold in carbon-nanotube-epoxy composites. Polymer.

[10] Adrianse L.J., Reedijk J.A., Teunissen P.A.A.H., Brom B., Michels M.A.J., Brokken-Zijp J.C.M. (1997). High-dilution carbon-black/polymer composites: Hierarchical percolating network derived from Hz to THz ac conductivity. Phys. Rev. Lett..

[11] Deng H., Lin L., Ji M., Zhang S., Yang M., Fu Q. (2014). Progress on the morphological control of conductive network in conductive polymer composites and the use as electroactive multifunctional materials. Prog. Polym. Sci..

[12] Bertasius P., Meisak D., Macutkevic J., Kuzhir P., Selskis A., Volnyanko E., Banys J. (2019). Fine tuning of electrical transport and dielectric properties of epoxy/carbon nanotubes composites via magnesium oxide additives. Polymers.

[13] Singh K., Ohlan A., Pham V.H., Balasubramaniyan R., Varshney S., Jang J., Hur S.H., Choi W.M., Kumar M., Dhawan S.K. (2013). Nanostructured graphene/Fe3O4 incorporated polyaniline as a high performance shield against electromagnetic pollution. Nanoscale.

[14] Luo X.L., Yang G.D., Schubert D.W. (2021). Electrically conductive polymer composite containing hybrid graphene nanoplatelets and carbon nanotubes: Synergistic effect and tunable conductivity anisotropy. Adv. Compos. Hybrid Mater..

[15] Gbaguidi A., Namilae S., Kim D. (2020). Synergy effect in nanocomposites based on carbon nanotubes and graphene nanoplatelets. Nanotechnology.

[16] Meisak D., Macutkevic J., Plyushch A., Kuzhir P., Selskis A., Banys J. (2020). Dielectric relaxation in hybrid Epoxy/MWCNT/MnFe_2_O_4_ composites. Polymers.

[17] Kumar R., Choudhary H.K., Anupama A.V., Menon A.V., Pawar S.P., Bose S., Sahoo B. (2019). Nitrogen doping as a fundamental way to enhance the EMI shielding behavior of cobalt particle-embedded carbonaceous nanostructures. New J. Chem..

[18] He Q.L., Yuan T.T., Zhang X., Luo Z.P., Haldolaarachchige N., Sun L., Young D., Wei S., Guo Z. (2013). Magnetically soft and hard polypropylene/cobalt nanocomposites: Role of maleic anhydride grafted polypropylene. Macromolecules.

[19] Hussain T., Ahmad M.N., Nawaz A., Mujahid A., Bashir F., Mustafa G. (2017). Surfactant incorporated Co nanoparticles with uniform dispersion and double percolation. J. Chem..

[20] Andreev A.S., Kazakova M.A., Ishchenko A.V., Selyutin A.G., Lapina O.B., Kuznetsov V.L., d’Espinose de Lacaillerie J.-B. (2017). Magnetic and dielectric properties of Carbon Nanotubes with embedded Cobalt nanoparticles. Carbon.

[21] Kazakova M.A., Semikolenova N.V., Korovin E.Y., Zhuravlev V.A., Selyutin A.G., Velikanov D.A., Moseenkov S.I., Andreev A.S., Lapina O.B., Suslyaev V.I. (2021). Co/Multi-walled carbon nanotubes/Polyethylene composites for microwave absorption: Tuning the effectiveness of electromagnetic shielding by varying the components ratio. Compos. Sci. Technol..

[22] Vasundhara K., Mandal B.P., Tyagi A.K. (2015). Enhancement of dielectric permittivity and ferroelectricity of a modiefied cobalt nanoparticle and polyvynilidene fluoride based composite. RSC Adv..

[23] Golubtsov G.V., Kazakova M.A., Selyutin A.G., Ishchenko A.V., Kuznetsov V.L. (2020). Mono-, bi- and trimetallic catalysts for the synthesis of multi-walled carbon nanotubes based on iron subgroup metals. J. Struct. Chem..

[24] Kazakova M.A., Koul A., Golubtsov G.V., Selyutin A.G., Ishchenko A.V., Kvon R.I., Kolesov B.A., Schuhmann W., Morales D.M. (2021). Nitrogen and oxygen functionalization of multi-walled carbon nanotubes for tuning bifunctional oxygen reduction/oxygen evolution performance of supported FeCo oxide nanoparticles. ChemElectroChem.

[25] Andreev A.S., Krasnikov D.V., Zaikovskii V.I., Cherepanova S.V., Kazakova M.A., Lapina O.B., Kuznetsov V.L., de Lacaillerie J.B.D.E. (2018). Internal field 59Co NMR study of cobalt-iron nanoparticles during the activation of CoFe2/CaO catalyst for carbon nanotube synthesis. J. Catal..

[26] Kazakova M.A., Andreev A.S., Selyutin A.G., Ishchenko A.V., Shuvaev A.V., Kuznetsov V.L., Lapina O.B., de Lacaillerie J.B.D.E. (2018). Co metal nanoparticles deposition inside or outside multi-walled carbon nanotubes via facile support pretreatment. Appl. Surf. Sci..

[27] Zhdanov A.A., Kazakova M.A. (2020). Use of Carbon Materials of Different Nature in Determining Metal Concentrations in Carbon Nanotubes by X-Ray Fluorescence Spectrometry. J. Anal. Chem..

[28] Kazakova M.A., Selyutin A.G., Semikolenova N.V., Ishchenko A.V., Moseenkov S.I., Matsko M.A., Zakharov V.A., Kuznetsov V.L. (2018). Structure of the in situ produced polyethylene based composites modified with multi-walled carbon nanotubes: In situ synchrotron X-ray diffraction and differential scanning calorimetry study. Compos. Sci. Technol..

[29] Kazakova M.A., Semikolenova N.V., Korovin E.Y., Moseenkov S.I., Andreev A.S., Kachalov A.S., Kuznetsov V.L., Suslyaev V.I., Mats’ko M.A., Zakharov V.A. (2018). In situ Polymerization Technique for Obtaining Composite Materials Based on Polyethylene, Multi-walled Carbon Nanotubes and Cobalt Nanoparticles. Russ. J. Appl. Chem..

[30] Kazakova M.A., Kuznetsov V.L., Semikolenova N.V., Moseenkov S.I., Krasnikov D.V., Matsko M.A., Ishchenko A.V., Zakharov V.A., Romanenko A.I., Anikeeva O.B. (2014). Comparative study of multiwalled carbon nanotubes/polyethylene composites produced via different techniques. Phys. Status Solidi B.

[31] Popok V.N. (2014). Polymer films with ion synthesized cobalt and iron nanoparticles: Conductance and magnetism. Rev. Adv. Mater..

[32] Plyushch A., Macutkevic J., Kuzhir P., Sokal A., Lapko K., Selskis A., Banys J. (2019). Synergy effects in electromagnetic properties of phosphate ceramics with silicon carbide whiskers and carbon nanotubes. Appl. Sci..

[33] Zeranska-Chudek K., Siemion A., Palka N., Mdarhi A., Elaboudi I., Brosseau C., Zdrojek M. (2021). Terahertz shielding properties of carbon black based polymer nanocomposites. Materials.

[34] Liu J., Duan C.G., Yin W.G., Mei W.N., Smith R.W., Hardy J.R. (2004). Large dielectric constant and Maxwell-Wagner relaxation in Bi_2/3_Cu_3_Ti_4_O_12_. Phys. Rev. B.

[35] Jonsher A.K. (1992). The universal dielectric response and its physical significance. EEE Trans. Electr. Insul..

[36] Almond D.P., Vainas B., Uvarov N.F. (1998). A new analysis of the bulk ac electrical response of ionic conductors. Solid State Ion..

[37] Sheng P. (1980). Fluctuation-induced tunneling conduction in disordered materials. Phys. Rev. B.

[38] Mdarhi A., Brosseau C., Zaghrioui M., El Aboudi I. (2012). Electronic conduction and microstructure in polymer composites filled with carbonaceous particles. J. Appl. Phys..

