# Supplementary material for: Dielectric Properties of Hybrid Polyethylene Composites Containing Cobalt Nanoparticles and Carbon Nanotubes
Source: Materials (Basel). 2022 Mar 2;15(5):1876. doi: 10.3390/ma15051876 (PMC8912063; doi:10.3390/ma15051876)
Supplement: Supplementary file 1 [file materials-15-01876-s001.zip › materials-1546871-supplementary.pdf]

materials

MDPI

Article

Dielectric Properties of Hybrid Polyethylene Composites Containing Cobalt Nanoparticles and Carbon Nanotubes

Ieva Vanskevičė [1], Mariya A. Kazakova [2], Jan Macutkevic [1,*], Nina V. Semikolenova [2] and Juras Banys [1]

[1] Faculty of Physics, Vilnius University, 10222 Vilnius, Lithuania; i.kranauskaite@yahoo.com (I.V.); juras.banys@ff.vu.lt (J.B.)

[2] Boreskov Institute of Catalysis, SB RAS, Lavrentieva 5, 630090 Novosibirsk, Russia; manj86@mail.ru (M.A.K.); nvsemiko@catalysis.ru (N.V.S.)

* Correspondence: jan.macutkievic@gmail.com; Tel.: +370-52234535

Citation: Vanskevičė, I.; Kazakova, M.A.; Macutkevic, J.; Semikolenova, N.V.; Banys, J. Dielectric Properties of Hybrid Polyethylene Composites Containing Cobalt Nanoparticles and Carbon Nanotubes. *Materials* **2022**, *15*, 1876. https://doi.org/10.3390/ma15051876

Academic Editor: Vlassios Likodimos and Bryan M. Wong

Received: 23 December 2021
Accepted: 26 February 2022
Published: 2 March 2022

Publisher's Note: MDPI stays neutral with regard to jurisdictional claims in published maps and institutional affiliations.

Copyright: © 2022 by the authors. Licensee MDPI, Basel, Switzerland. This article is an open access article distributed under the terms and conditions of the Creative Commons Attribution (CC BY) license (https://creativecommons.org/licenses/by/4.0/).

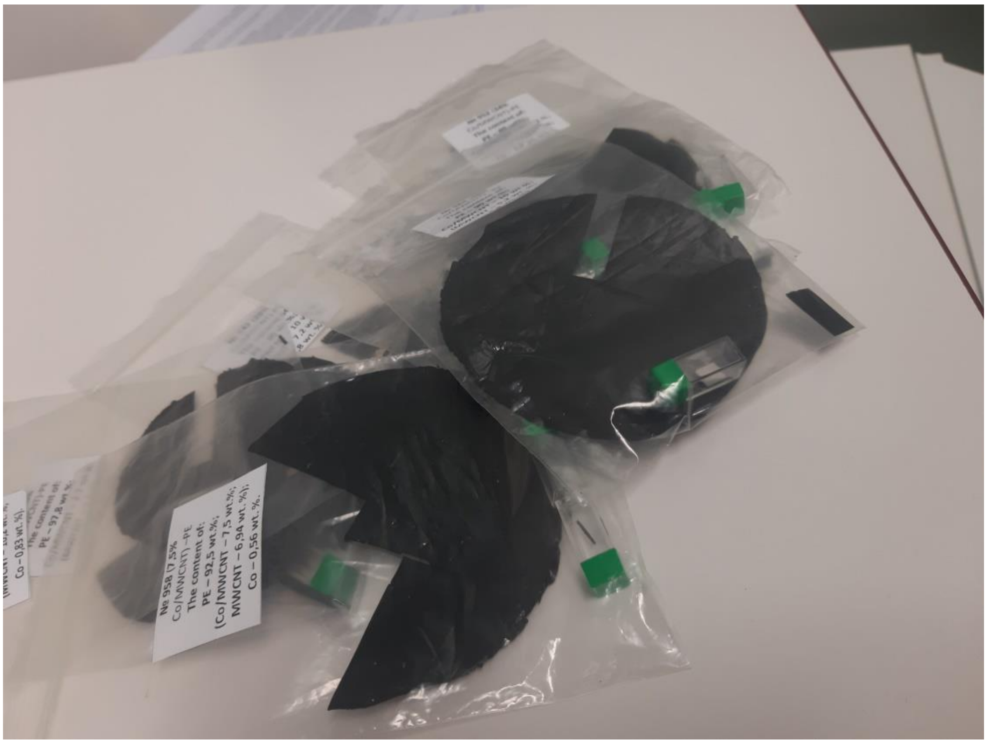

Figure S1. Investigated composites photos.

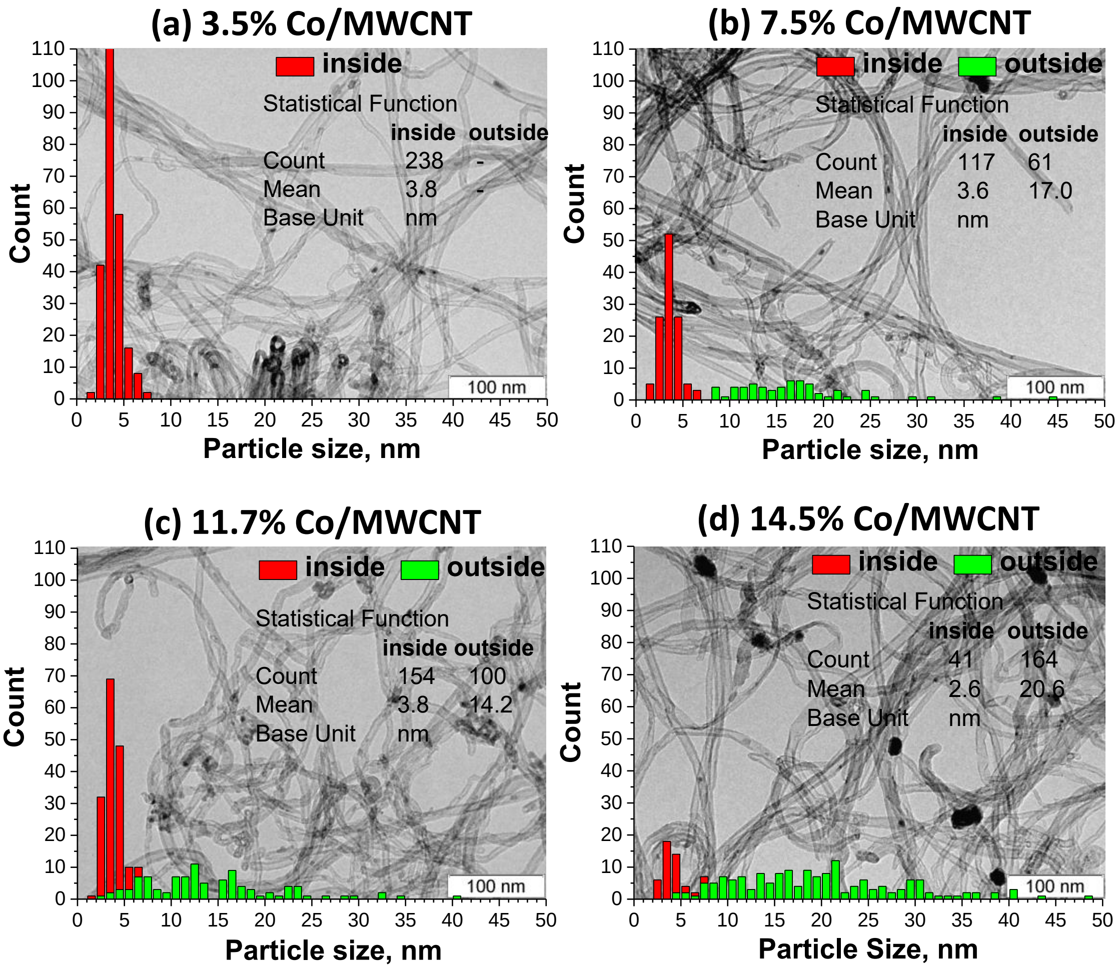

Figure S2. TEM images and Co nanoparticles size distribution in 3.5–14.5% Co/MWCNT hybrids (**a**–**d**) prepared by one step incipient wetness impregnation.

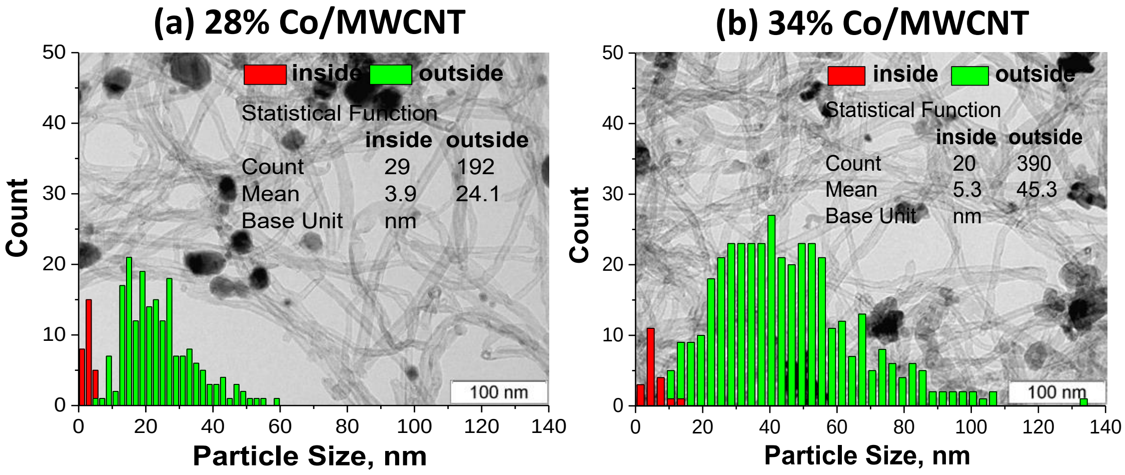

Figure S3. TEM images and Co nanoparticles size distribution in 28–34% Co/MWCNT hybrids (**a**,**b**) prepared by multiple incipient wetness impregnation.

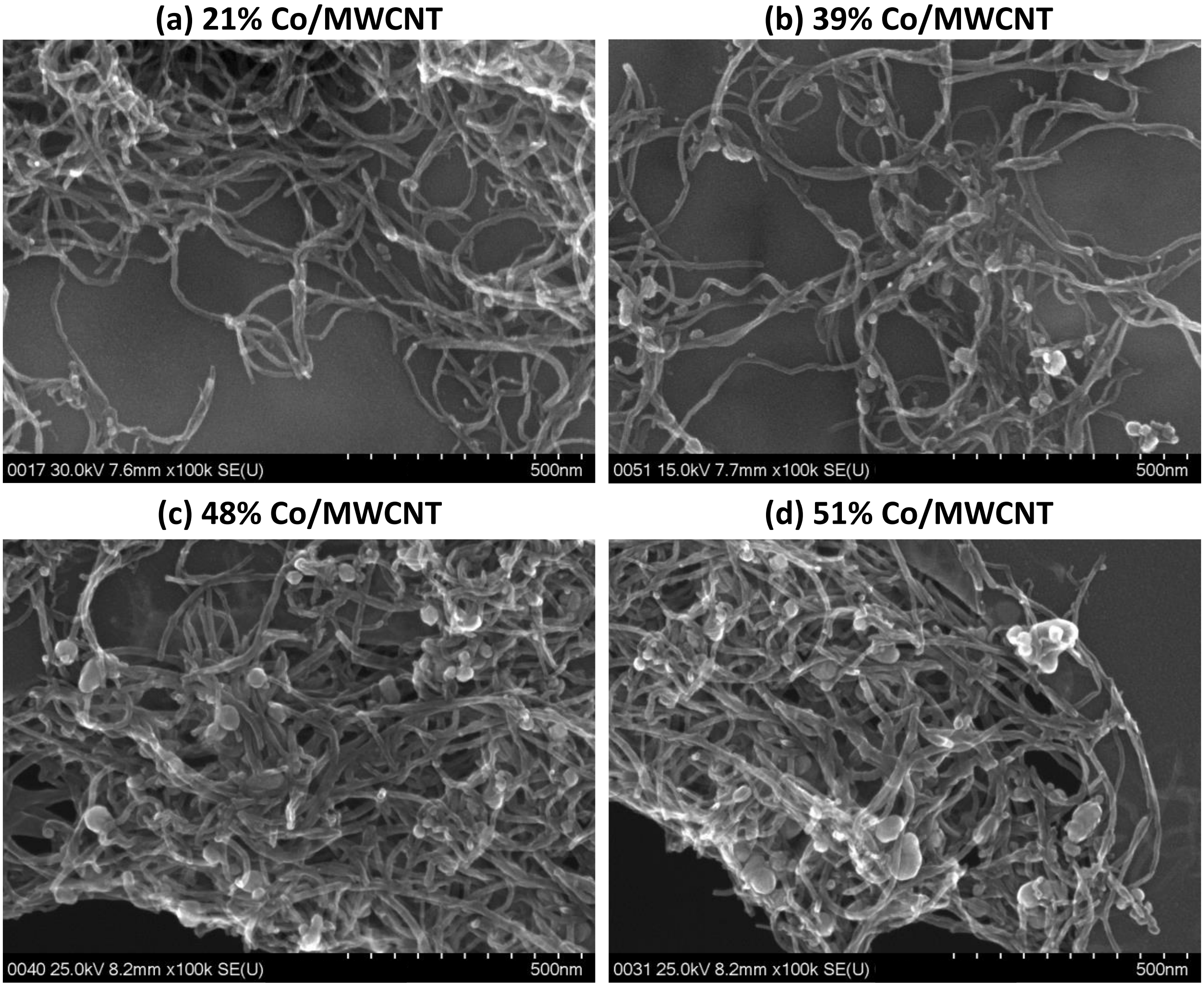

Figure S4. SEM images for 21–51% Co/MWCNT hybrids (**a**,**b**) prepared by multiple incipient wetness impregnation.
